# Neutrophil/Lymphocyte Ratios, Platelet/Lymphocyte Ratios, and Mean Platelet Volume Values in Patients with Measles

**DOI:** 10.7759/cureus.6607

**Published:** 2020-01-09

**Authors:** Abdullah Solmaz, Abit Demir, Hüseyin Gümüş, Mustafa Aksoy, Filiz Solmaz

**Affiliations:** 1 Pediatrics, Harran University, Şanlıurfa, TUR; 2 Dermatology, Harran University, Şanlıurfa, TUR

**Keywords:** measles, neutrophil/lymphocyte ratios, platelet/lymphocyte ratios, mean platelet volume

## Abstract

Aim: The purpose of this study was to compare neutrophil/lymphocyte ratio (NLR), platelet/lymphocyte ratio (PLR), and mean platelet volume (MPV) values of patients with measles within the healthy control group and then determine the utility of these parameters for determination of inflammatory situation in patients with measles.

Materials and method: A total of 51 pediatric patients who had visited Harran University Medical Faculty Pediatrics Clinic between June 2018 and May 2019 and who had been diagnosed with measles after anamnesis, clinical, and serological evaluations were included our study; 49 healthy children who visited our clinic for various reasons and had taken hemogram tests were also added to our study as the control group.

Results: NLR and PLR values were observed higher than the control group for patients diagnosed with measles; however, the difference between the two groups was not statistically significant (p values 0.515 and 0.796, respectively). When MPV values of patients diagnosed with measles and control groups were compared, it was determined that MPV value was statistically lower in patients diagnosed with measles (p: 0.001).

Conclusion: Based on the parameters obtained through our study, it can be said that NLR and PLR are not suitable parameters for proving inflammation in patients with measles but MPV can be used as a convenient parameter for that purpose. However, prospective studies conducted with more patients are needed in this respect.

## Introduction

Measles is particularly seen during childhood; it is an epidemic and a highly contagious infectious disease. Its agent is the rubeola virus which is a part of the Paramyxoviridae family. The spread of the disease occurs as a result of aerosol-form parts of the respiratory secretions of patients reaching the sensitive host. For the infection of measles, both antibody response and cellular response is important [1-4]. Hematological side effects such as leucopenia, lymphopenia, and thrombocytopenia are observed to be connected with measles infection [5-6].

Even though various identifiers have been defined for many diseases during the recent years, the determination of these identifiers takes time and is costly. Peripheral blood cells display quantitative and functional changes as a response to the inflammation evolving in the body [7]. Based on these changes, a series of inflammatory biological identifiers like neutrophil/lymphocyte ratio (NLR), platelet/lymphocyte ratio (PLR), and mean platelet volume (MPV), which could be easily obtained through peripheral blood count in infectious or inflammatory diseases, were examined [8-10].

There is no such study in the literature that evaluates the inflammatory parameters like NLR, PLR, and MPV in patients with measles. The purpose for this study is to compare the NLR, PLR, and MPV values of patients with measles within the healthy control group and then determine the utility of these parameters for the determination of inflammatory situations in patients with measles.

## Materials and methods

A total of 51 pediatric patients who had visited Harran University Medical Faculty Pediatrics Clinic between June 2018 and May 2019 and who had been diagnosed with measles after anamnesis, clinical, and serological evaluations were included our study; 49 healthy children who visited our clinic for various reasons and had taken hemogram tests were also added to our study as the control group; they did not have any viral, bacterial, and fungal infection, systemic inflammatory disease or malignancy in any organs.

The gender, age, neutrophil count, lymphocyte count, leucocyte count, platelet count, NLR, PLR, and MPV values of the patients and the control group were retrospectively scanned and recorded. NLR was calculated by dividing the neutrophil count by the lymphocyte count and PLR was calculated by dividing the platelet count by the lymphocyte count. The complete blood count (CBT) was calculated through the optical scatter laser method (Cell-Dyn 3700, Abbott Diagnostics, Chicago, USA).

The exclusion criteria for the study were determined as any thyroid disease, cardiovascular disease, hypertension, hypercholesterolemia, systemic diseases like diabetes mellitus, malignancy in any organs, or existence of a local or systemic infection.

Data distributed check was conducted using the Shapiro-Wilk test. Since the data distribution was normal, the student t-test was used for the comparison of the two groups. Identifying statistics for continuous variables are shown as mean and standard deviation values. For the analysis of genders, the Pearson chi-square was used. Statistical analysis was performed using the Statistical Package for the Social Sciences; version 21 (SPSS Inc., Chicago, IL) program; p<0.05 was accepted as statistically significant.

## Results

For our study, any statistically significant difference was not detected in terms of gender and age ratio between patients diagnosed with measles (26 males, 25 females; mean±sd age 5.42±2.31) and the healthy control group (26 males,23 females; mean±sd age 5.41±3.41) (p>0.05).

The neutrophil count of the patients diagnosed with measles was found as 4.31±2.83 x 103 and the neutrophil count of the healthy control group was determined as 5.87±2.41 x 103, and the difference between two groups was statistically significant (p:0.001).

The lymphocyte count of the patients diagnosed with measles was found as 3.27±2.34 x 103 and the lymphocyte count of the healthy control group was determined as 3.28±1.01 x 103, and the difference between two groups was not statistically significant (p:0.339).

The leukocyte count of the patients diagnosed with measles was found as 8.57±4.31 x 103 and the leukocyte count of the healthy control group was determined as 10.17±3.08 x 103, and the difference between two groups was statistically significant (p:0.005).

The platelet count of the patients diagnosed with measles was found as 325.25±158.31 x 103 and the platelet count of the healthy control group was determined as 384.96±110.65 x 103, and the difference between two groups was statistically significant (p:0.002).

The NLR value of the patients diagnosed with measles was found as 2.41±2.43 and the NLR value of the healthy control group was determined as 1.94±0.86, and the difference between the two groups was not statistically significant (p: 0.515).

The PLR value of the patients diagnosed with measles was found as 167.57±145.58 and the PLR value of the healthy control group was determined as 124.43±30.16, and the difference between two groups was not statistically significant (p:0.796).

The MPV value of the patients diagnosed with measles was found as 6.42±1.38 and the MPV value of the healthy control group was determined as 7.03±0.92, and the difference between the two groups was statistically significant (p:0.001).

The laboratory results of the patients diagnosed with measles and the control group are presented in Table 1.

**Table 1:** Laboratory findings of patients with measles and the healthy control group

Parameters	Measles (n:51)(Mean ± SD)	Healthy control (n:49)(Mean ± SD)	P value
Number of neutrophils (x10^3^/ml)	4.31±2.83	5.87±2.41	0.001
Number of lymphocyte (x10^3^/ml)	3.27±2.34	3.28±1.01	0.339
Number of leukocyte (x10^3^/ml)	8.57±4.31	10.17±3.08	0.005
Number of platelet (x10^3^/ml)	325.25±158.31	384.96±110.65	0.002
NLR	2.41±2.43	1.94±0.86	0.515
PLR	167.57±145.58	124.43±30.16	0.796
MPV	6.42±1.38	7.03±0.92	0.001
NLR: Neutrophil/lymphocyte ratio, PLR: Platelet/lymphocyte ratio, MPV: Mean platelet volume (Student-T test)			

## Discussion

During the incubation period, the measles virus settles in the regional lymphoid tissues. The first viremia occurs in the reticuloendothelial system and the second viremia occurs at the body surface. Viral distribution starts with prodromal symptoms. Once the antibody production starts along with rashes, then viral distribution and symptoms decrease simultaneously. Immune system suppression occurs through the suppression of Th1 cells by infecting CD4+ T cells. The studies conducted during the recent years have shown that the measles virus uses nectin-4 receptors in the respiratory tract epitheliums by targeting alveoli macrophages, dendritic cells, and lymphocytes; for other tissues, it uses CD150 receptors on which the measles virus performs lymphocyte stimulation [11-14].

The disease is first defined with clinical findings; the presence of lymphopenia supports the findings. If the measles is not epidemic, the disease is diagnosed clinically. Serologically, it is expected that the measles IgM levels start to increase from day one or two of the disease up to the end of a month. However, if the measles IgM, which is taken during the first 72 hours of the disease, is negative, it might need to be repeated two-four weeks after the acute disease sets in. Four times or more increase in IgG level indicates the diagnosis. Production of the virus through culturing blood, urine, and respiratory tract samples and proving the virus itself or viral ribonucleic acid with polymerase chain reaction (PCR) technique is possible [15-17].

The white blood cells are the main cells of inflammation and play a role in the pathogenesis of many diseases. An increase in neutrophil counts during the acute inflammatory processes, along with decrease in lymphocyte counts based on acute stress, reflects the changes in the immune system. Existence of leucocytosis is interpreted in support of inflammation or infection [18-20]. In recent years, a value that includes both increasing neutrophil counts during the acute inflammation and the decreasing lymphocyte count during acute stress has been utilized. This value is called as NLR and its popularity is increasing day by day. NLR value is generally accepted as the indication of subclinical inflammation and it has been observed that this value increases during many infectious and inflammatory diseases [10,21-22].

The evolution of leucopenia in 12 patients has been observed during the study conducted by Karakecili et al. with 28 adult patients with measles [5]. Another study evaluating adult patients with measles conducted by Sunnetcioglu et al. has observed leucopenia in 7 (14%) patients [6]. Leucopenia evolving following the measles infection is accepted as secondary in bone marrow suppression and is related to apoptosis in non-infected cells [13]. There is no such study in the literature that evaluates NLR parameters in patients with measles. In our study, NLR values of patients with measles was higher than the control group, and the difference between the two groups was not statistically significant.

Platelets are blood cells that play a role in coagulation. In case of any bleeding, the immediately reach that affected area and try to stop the bleeding by sticking to each other and constricting the vein. As being a part of the natural immune system, platelets could be increased as a response to “acute phase reaction” during the inflammation process. This increase in platelets may reflect the activity of bone marrow cells as a response to inflammatory phase interleukins such as IL-1 and Il-6 [23-24]. Large platelets are metabolically more active. MPV is the most commonly used measurement of platelet size and is accepted as in-vivo sign of the reactivity of platelets [9,25].

Thompson et al. have determined the relation between the MPV and platelet aggregation, positive correlation between ATP in platelets and β thromboglobulin content and that ATP and β thromboglobulin secretion progressively increases along with the increase in mean platelet volume after the stimulation [25]. Consequently, MPV alone could be accepted as an indicator of platelet activation [26]. Many studies taken place in the literature have reported the relation between MPV and many inflammatory and infectious diseases [9,27].

Thrombocytopenia was observed in 26 (52%) patients in the study conducted by Sunnetcioglu et al. with 50 adult patients with measles [6]. However, as far as we know, there is no such study in the literature that evaluates MPV parameters in patients with measles. In our study, the MPV values of patients with measles were lower than the control group, and the difference between two groups was statistically significant.

In recent years, it has been proved that the PLR value, obtained through dividing platelet count to lymphocyte count, has an important effect on systemic inflammatory response pathogenesis and can be used as an inflammation indicator [8,28]. Many studies in the literature have reported the relation between PLR (as well as MPV) and many inflammatory and infectious diseases [9,29-30]. Even though thrombocytopenia is observed in patients with measles, there is no such study in the literature that evaluates PLR levels in patients with measles. In our study, PLR values of patients with measles were higher than the control group, and the difference between the two groups was not statistically significant.

## Conclusions

Based on the data obtained through our study, it has been concluded that NLR and PLR are not suitable parameters for proving inflammation in patients with measles, but MPV is an appropriate parameter in terms of proving the inflammation in patients with measles. However, prospective studies conducted with more patients are needed in this respect.
